# Altitude and human disturbance are associated with helminth diversity in an endangered primate, *Procolobus gordonorum*

**DOI:** 10.1371/journal.pone.0225142

**Published:** 2019-12-04

**Authors:** Claudia Barelli, Viviana Gonzalez-Astudillo, Roger Mundry, Francesco Rovero, Heidi C. Hauffe, Thomas R. Gillespie

**Affiliations:** 1 Department of Biodiversity and Molecular Ecology, Research and Innovation Centre, Fondazione E. Mach, San Michele all’Adige, Trento, Italy; 2 MUSE–Science Museum, Tropical Biodiversity Section, Trento, Italy; 3 Department of Environmental Sciences and Program in Population Biology, Ecology and Evolutionary Biology, Emory University, Atlanta, GA, United States of America; 4 Pathology Resident, California Animal Health & Food Safety Laboratory, University of California, Davis, CA, United States of America; 5 Max Planck Institute for Evolutionary Anthropology, Leipzig, Germany; 6 Department of Biology, University of Florence, Florence, Italy; 7 Department of Environmental Health, Rollins School of Public Health, Emory University, Atlanta, GA, United States of America; Universidad Nacional Autonoma de Mexico Instituto de Investigaciones en Ecosistemas y Sustentabilidad, MEXICO

## Abstract

Gastrointestinal parasites colonizing the mammalian gut influence the host immune system and health. Parasite infections, mainly helminths, have been studied intensively in both humans and non-human animals, but relatively rarely within a conservation framework. The Udzungwa red colobus monkey (*Procolobus gordonorum*) is an endangered endemic primate species living in the Udzungwa Mountains of Tanzania, a global biodiversity hotspot. Since this endemic primate species is highly sensitive to human disturbance, here we investigate whether habitat type (driven by natural and human-induced factors) is associated with helminth diversity. Using standard flotation and sedimentation techniques, we analyzed 251 fecal samples belonging to 25 social groups from four different forest blocks within the Udzungwa Mountains. Five parasitic helminth taxa were recovered from Udzungwa red colobus, including *Trichuris* sp., *Strongyloides fulleborni*, *S*. *stercoralis*, a strongylid nematode and *Colobenterobius* sp. We used Generalized Linear Mixed Models to explore the contribution of habitat type, altitude and fecal glucocorticoid levels (as biomarkers of stress) in predicting gut parasite variation. Although some parasites (e.g., *Trichuris* sp.) infected more than 50% of individuals, compared to others (e.g., *Colobenterobius* sp.) that infected less than 3%, both parasite richness and prevalence did not differ significantly across forests, even when controlling for seasonality. Stress hormone levels also did not predict variation in parasite richness, while altitude could explain it resulting in lower richness at lower altitudes. Because human activities causing disturbance are concentrated mainly at lower altitudes, we suggest that protection of primate forest habitat preserves natural diversity at both macro- and microscales, and that the importance of the latter should not be underestimated.

## Introduction

Gastrointestinal parasitic worms (mainly helminths) represent the most prevalent infectious agents affecting nearly one-third of the human population, as well as most livestock and wildlife, especially in the tropics and in developing countries [1, 2, 3]. Although helminths cause considerable human morbidity and mortality worldwide [4] with a notable economic impact, their absence in the gut biota of humans consuming a ‘western’ diet has been widely associated with an increased prevalence of auto- and hyper- immune diseases [5, 6]. This suggests that helminths are an essential player in host gut homeostasis and health. While it is widely accepted that human activities causing habitat disturbance or loss (especially encroaching agriculture and logging) lead to loss in species diversity at a macro scale [7], we know far less about the impact of these activities on biodiversity at a micro-scale, such as that of the gastrointestinal tract.

Animals living in fragmented and disturbed habitats are more likely to experience habitat saturation leading to higher population density, reduced food availability and elevated stress hormone levels (leading to lowered immune system function) [8, 9]; all factors which facilitate the transmission of helminths [10, 11, 12]. Some studies indicate that the proportion of infected hosts (parasite prevalence) and/or the number of different parasite taxa infecting each host (parasite richness) increase with increasing habitat disturbance [11, 13–16]. However, opposite associations [17, 18; 19] or no relationships have also been found [20].

Tropical non-human primates live in pristine forests that are constantly reduced by human activities, making primates one of the most threatened taxa on the planet [21]. Exploring whether human-induced habitat changes may affect also biodiversity at micro scale, such as that of gastrointestinal parasites, is urgent and relevant for the plausible implications on animal health and conservation. We addressed this question in the Udzungwa red colobus (*Procolobus gordonorum*), an endangered primate endemic to the forests of the Udzungwa Mountains (hereafter Udzungwas) in south-central Tanzania. The area is of critical importance for biological diversity and endemism [22, 23, 24]. However, it is also increasingly threatened by intensive agriculture [25], subsistence logging and harvesting [21, 24] making primate populations particularly vulnerable to hunting [26, 27]. Forests within the Udzungwas are currently fragmented into numerous blocks, widely different in habitat structure and exposure to anthropogenic disturbance (from national park to forest reserve or completely unprotected). Because the climate of the area shows seasonal variations and the study sites vary widely in altitude gradient (within and among study sites), natural environmental factors need to be also taken into account when predicting parasite infections [28–32]. Hence, we here explore the variation in parasite prevalence and richness in the endemic and endangered Udzungwa red colobus living in natural and modified habitats.

## Materials and methods

### Study site

**Fig 1:**
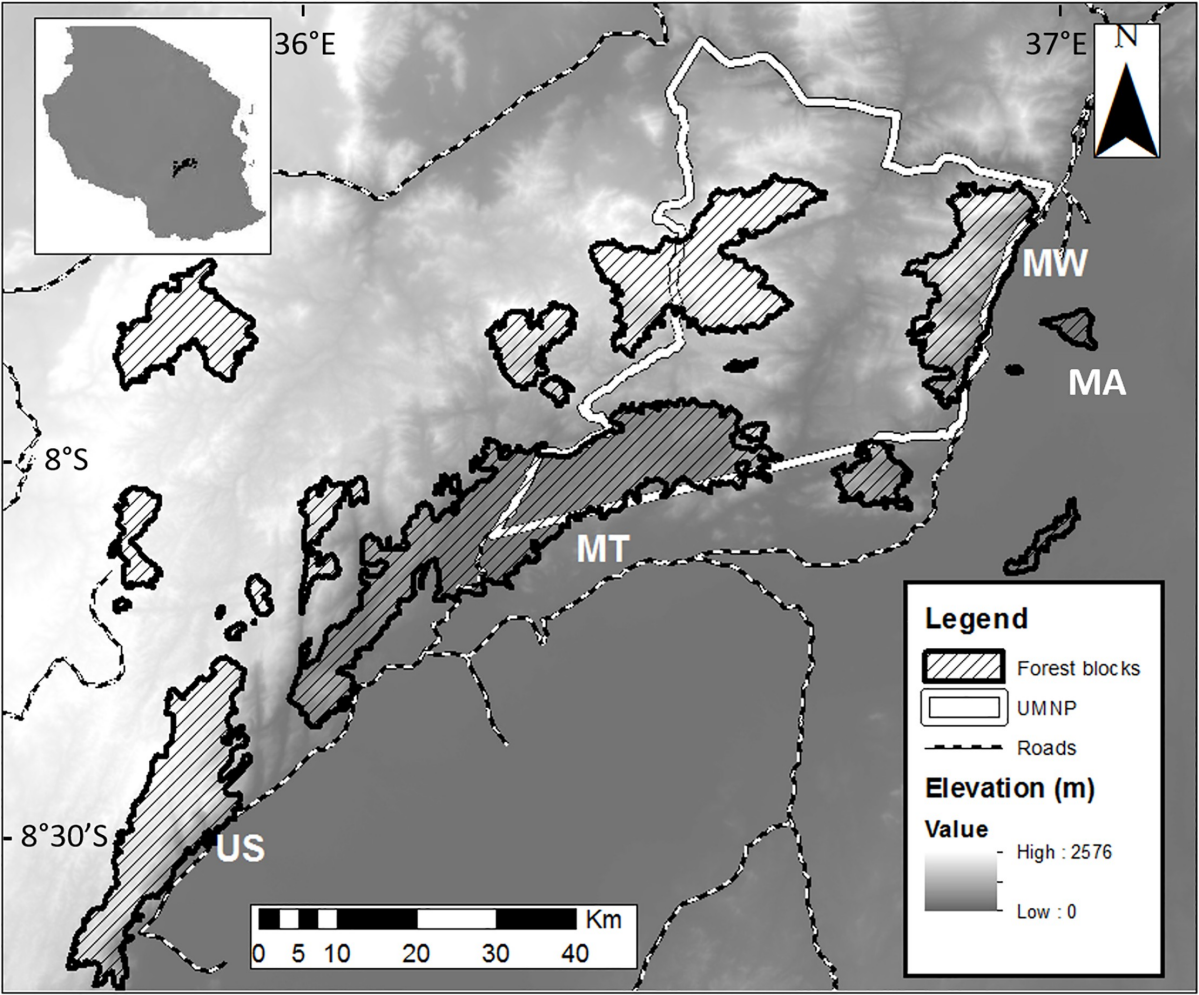
Map of the study sites. Map of the Udzungwa Mountains of Tanzania showing the four forest blocks sampled (MA: Magombera, MT: Matundu, MW: Mwanihana, US: Uzungwa Scarp Nature Reserve). The borders of the Udzungwa Mountain National Park (UMNP) are highlighted in white. (Original map from [33]).

The Udzungwas represent the southernmost mountain block in the Eastern Arc Mountains and occupy an area of approximately 19,000 km^2^ (7°40’ S to 8°40’ S and 35°10’ E to 36°50’ E; Fig 1). Average annual rainfall varies from 1,500 to 2,000 mm per year, distributed into two main seasons: November-December and March-May.

Social groups of the endangered and endemic Udzungwa red colobus monkeys were studied in four forest blocks: Magombera (MA), Uzungwa Scarp Nature Reserve (US), Matundu (MT) and Mwanihana (MW). Long-term studies on such forest blocks have already assessed and revealed differences in habitat structure, vegetation parameters and human disturbance, highlighting plausible associations between both primate population density and their genetic diversity to habitat type and human-driven changes [Table 1; 34, 35,36]. Moreover, all forest blocks have been separated from each other for over 60 years by at least 6 km (longer than the maximum dispersal range of the study species), with urbanized or agricultural areas concentrated at lower altitude or at forest edges [35].

**Table 1:** Characteristics of the four study forests in the Udzungwa Mountains of Tanzania (MA, US, MT, MW) ordered by the degree of human impact and level of protection, from the most disturbed and least protected (MA) to the least disturbed and most protected (MW).

Forest	Area (km^2^)	Altitude (m a.s.l.)	Density (groups/km^2^) (SE)	Habitat structure, protection level and human activities
Magombera (MA)	11.9	269–302	4.88 (0.97)	Ground-water lowland evergreen forest; one of the few remaining patches of once continuous lowland forest; surrounded by villages and intensive agriculture. No formal protection; the forest is shrinking in size and frequently encroached for firewood collection, pole cutting and hunting
Uzungwa Scarp (US)	314.5	290–2,144	1.2 (0.34)	Lowland, semi-deciduous, sub-montane and montane evergreen forest, including upper montane, bamboo-dominated forest. Nature Reserve since 2016; no resource extraction allowed, but pole and timber cutting, as well as hunting are illegally practiced; several villages along the border
Matundu (MT)	526.3	279–1,046	2.4 (0.41)	Lowland to sub-montane evergreen to deciduous forest, with large portions logged in the past, now secondary, regenerating vegetation with low tree diversity. National Park since 1992, with a smaller portion (unsampled in this study) falling in Kilombero Nature Reserve.
Mwanihana (MW)	150.6	351–2,263	1.83 (0.33)	Continuous forest escarpment, similar to US. National Park since 1992; several villages along the eastern edge.

Data on forest area and altitude from [36, 37].

#### Sample collection and parasitological analyses

In order to avoid disturbing this shy and elusive species, from July 2011 to October 2012, four trained field assistants followed social groups from a distance of approximately 10–15 meters as they moved through the forest canopy, and collected fecal samples for parasitological analysis opportunistically. Since fecal samples could not be assigned to a specific individual or sex, to avoid any potential re-sampling, samples were collected during a single defecation event from each group, and sampled groups were at least 2 km apart (N = 25 groups and 251 fecal samples; S1 Table). Sampling procedures are described in more detail in [35, 38, 39]. All samples were examined macroscopically for consistency, and presence of blood, mucus, tapeworm proglottids and adult and larval nematodes. An aliquot of 2 g of fresh feces was placed in a polypropylene tube containing 10 ml of 10% neutral buffered formalin and stored it at ambient temperature (20–25°C) until shipment to Emory University, USA for parasitological analysis.

The eggs and larvae of metazoan parasites were recovered via sodium nitrate flotation and fecal sedimentation utilizing standard techniques as previously described [40]. Slides were examined under a compound microscope at 400X magnification, and parasite identification was performed on the basis of the size, shape, and contents of the egg and larvae. If needed, a drop of Lugol’s iodine solution was added to the sample to assist in parasite identification. Representative examples were measured to the nearest 0.1 μm with an ocular micrometer with Leica computer software. Parasite quantification was carried out by examining one slide per sample as previously described [41], by removing the cover slip from the flotation tube and placing it on a slide. We scanned slide using the × 10 objective lens of a compound microscope and identified and counted all parasite eggs and larvae. We then used the × 40 objective lens for measurement and confirmation of identifications. We measured the length and width of individual eggs and larvae using a calibrated ocular micrometer and photographed representatives.

#### Assessment of stress hormone levels

To assess the link between physiological stress and parasite infections, fecal glucocorticoid (FGC) levels were determined for a subset of 202 samples collected from 20 groups between 8h00 and 10h00 directly following defecation (S1 Table). Fresh feces were homogenized with gloved hands, and any obvious undigested matter was removed. For each sample, approximately 0.5 g of stool was placed in a 15ml polypropylene tube pre-filled with 4 ml of 80% ethanol, and the tube was shaken manually for 30 seconds to produce a fecal suspension [38, 42, 43]. We used Parafilm (Pechiney Plastic Packaging Company, USA) to avoid evaporation and leakage until return to the research station where we kept all samples in the dark at room temperature for 15 days before extraction. All samples underwent exactly the same duration of storage in alcohol, eliminating any potential storage-time dependent variation in FGC levels [44]. Prior to extraction, tubes containing the fecal suspension were weighed to determine fecal wet weight to the nearest 0.001g, and fecal samples were extracted following established procedures [43, 45]. In summary, the tube containing the fecal suspension was shaken firmly for 2 minutes, centrifuged at high speed for 2 min using a manually operated centrifuge (Hettich GmbH & Co. KG Tuttlingen, Germany). Two ml of the supernatant (containing the dissolved steroids) was pipetted into a 2 ml polypropylene tube (PPT; SafeSeal Micro Tube; Ref. No. 72.695.200 from Sarstedt AG & Co. Nuernbrecht, Germany), and sealed them with Parafilm and stored them in the dark at ambient temperature (20–25°C). The latter procedure has been shown to be effective for storing fecal extracts long-term without affecting FGC levels [46, 47].

#### Ethics statement

The authors confirm they did not interact with the red colobus social groups in any way. Fecal sample collection was conducted using non-invasive methods and adhered to the ‘Code of Best Practices for Field Primatology’ of the International Primatological Society (IPS) and to the ‘Principles for the Ethical Treatment of Primates’ of the American Society of Primatologists (ASP). The study was conducted with permission to CB and FR from the Tanzania Commission for Science and Technology (COSTECH Permits No. 2011-85-NA-2011-33; 2011-84-NA-2011-33; 2011-351-NA-2011-68; 2011-346-NA-2011-183), Tanzania Wildlife Research Institute (TAWIRI) and Tanzania National Parks (TANAPA). All applicable institutional and/or national guidelines for the care and use of animals were followed.

### Data analyses

#### Comparing parasite prevalence among forests (model 1)

To compare parasite prevalence (number of hosts infected with one or more individuals of a particular parasite taxon in each fecal sample [48, 49] (for definition) among forests, we used a Generalized Linear Mixed Model (GLMM) [50], one for each parasite taxon, with binomial error structure and logit link function [51, 52]. As response variables we used parasite prevalence per fecal sample for the three most common taxa (*Trichuris* sp., *Strongyloides fulleborni*, strongylid nematode) separately, because the other two (*S*. *stercoralis* and *Colobenterobius* sp.) were found in only two and four samples respectively. We included forest block as a fixed effect, and social group as a random intercepts effect. To further account for potential seasonal variation in parasite load, we also included the sine and cosine of Julian date (after turning it into radians by first dividing by 365.25 and then multiplying by 2 × π) [53]. The data for these models included 211 fecal samples from 21 groups.

#### Comparing parasite richness among forests (model 2)

Similarly, we compared parasite richness (number of different parasite types per sample) among forests by using a GLMM with binomial error structure and logit link function and with the same fixed and random effects used in model 1. However, we included one additional random effect for the sample ID to account for the non-independence of parasite presence and absence from the same sample. This model essentially modeled the proportion of all five parasite taxa found per sample, including samples in which no parasites were found. Overdispersion was also not an issue (dispersion parameter = 0.838) and data comprised 251 fecal samples from 25 groups.

#### Comparing parasite prevalence and richness among groups within forests

To compare parasite prevalence among groups of the same forest, we considered the variance estimated for the contribution of group in the above models (for reasons of comparability with the estimates obtained for the fixed effects, we report the respective standard deviations). We do not provide a formal significance test because the degrees of freedom associated with an estimated random effect are currently not known [54].

#### Parasite richness as a function of altitude and FGC level (model 3)

This model estimated the proportion of possible parasites a sample was infected with as a function of altitude or FGC levels (i.e., it was a GLMM fitted with binomial error structure and logit link function). As fixed effects predictors, we included forest, FGC levels per group (averaged after square root transformation), altitude (square root transformed after subtracting the minimum altitude) per group, and season as described for model 1. Furthermore, we included random intercepts effects for group and sample ID. As an overall test of the effect of altitude and stress hormone level, we compared this full model with a null model lacking these factors but being otherwise identical [55]. The predictors date and altitude were moderately collinear with one another and with population [56, 57] with a maximum Generalized Variance Inflation Factor (GVIF; taken to the power of 1/twice the degrees of freedom of the respective effect, then squared) of 5.51 [58]; however, a visual inspection of Julian date plotted against altitude indicated that there was sufficient variation in both across their ranges. Furthermore, both altitude and sample date varied considerably within populations. Hence, model results are unlikely to suffer from collinearity. Overdispersion was no issue (dispersion parameter = 0.843). The data for this model comprised 202 samples taken from 20 groups.

#### Parasite prevalence as a function of altitude and stress hormones (model 4)

These models were identical to model 1 with the exception that we added altitude and stress hormone levels as described in model 3. The data used for all these models comprised 162 samples taken from 16 social groups. Collinearity among predictors was not evident (maximum GVIF taken to the power of 1/twice the degrees of freedom of the respective effect and then squared = 6.186; see model 3), nor was overdispersion (range of dispersion parameters: 0.761 to 0.938). We conducted a full-null model comparison as described for model 3.

#### General considerations

We conducted the analyses in R (version 3.5.3) [59]. For GLMMs we used the function ‘glmer’ in the package lme4 (version 1.1–21) [60] or glmmTMB (version 0.2.3 from the package glmmTMB (0.2.3) [61] in case of models with a negative binomial error distribution. We determined Generalized Variance Inflation Factors using the function ‘vif’ of the package car [62]. We estimated model stability by excluding groups one at a time and comparing model estimates obtained for the subsets with those obtained for the full data set. We used a parametric bootstrap to obtain confidence intervals of model estimates (function bootMer of the package lme4 or simulate.glmmTMB of the package glmmTMB). Throughout we determined the significance of individual effects or groups of terms by using likelihood ratio tests [63] comparing a respective full model with a reduced model lacking the effect(s) to be tested [64]. The reason that we fitted models 3 and 4 in addition to models 1 and 2 rather than including altitude and stress hormone levels into model 1 and 2 was that we did not have FGC levels for all samples but wanted to use a sample as large as possible for the group comparisons.

#### Results

**Fig 2:**
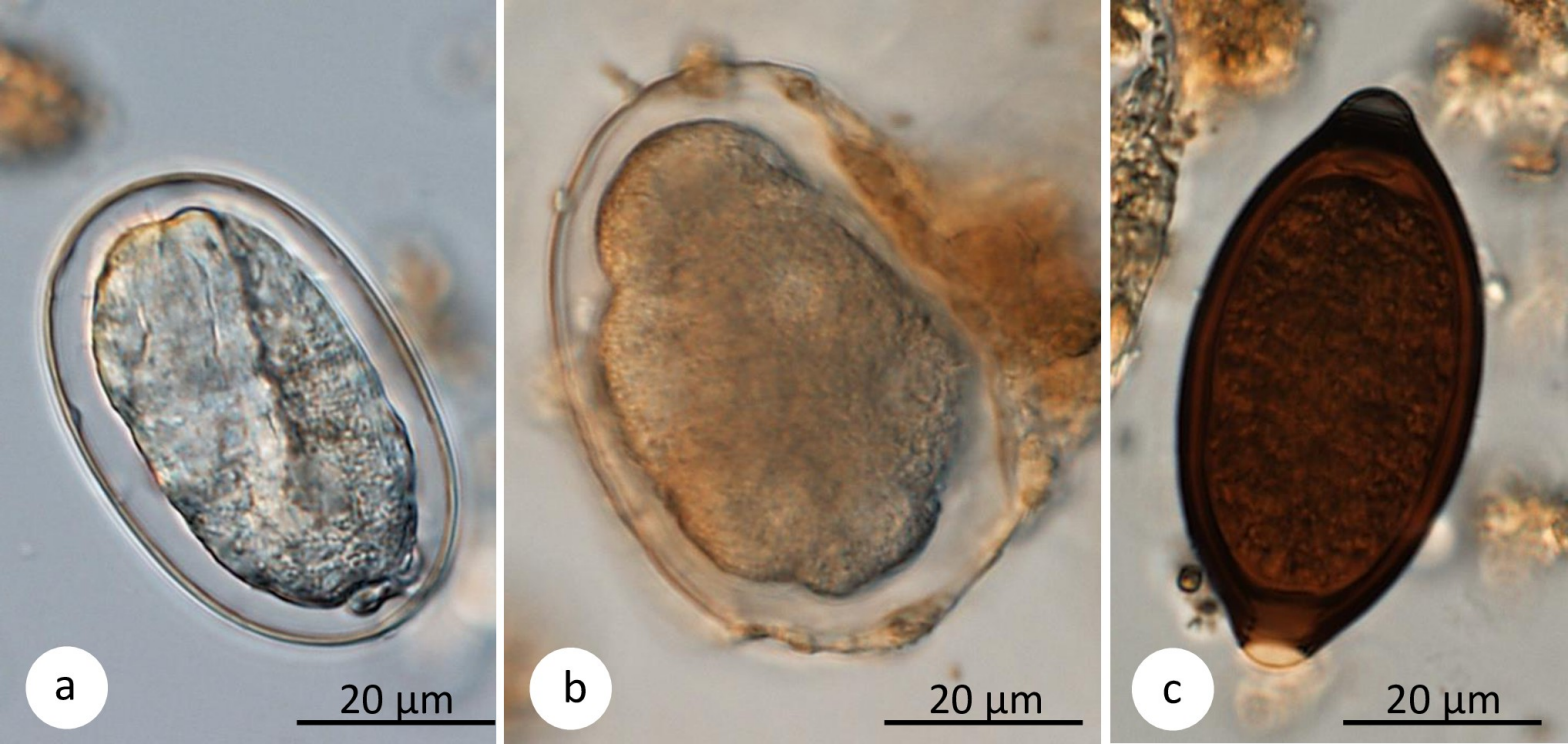
Parasite eggs. Sample pictures of gastrointestinal parasites found by sedimentation and flotation methods in Udzungwa red colobus monkeys: (a) *Strongyloides fulleborni*, (b) strongylid nematode, (c) *Trichuris* sp. Scale bars: 20 μm.

Five different gastrointestinal parasites were recovered from fecal samples of the Udzungwa red colobus monkeys: *Trichuris* sp., *Strongyloides fulleborni*, *S*. *stercoralis*, *Colobenterobius* sp. and one strongylid nematode (egg morphology did not permit species identification; Table 2; Fig 2). We identified at least one nematode species in 72% (181/251) of fecal samples.

**Table 2:** Overall prevalence (expressed in %) of gastrointestinal parasites in the endemic and endangered Udzungwa red colobus monkeys collected in four forests within the Udzungwa Mountains of Tanzania (MA: Magombera, US: Uzungwa Scarp Nature Reserve, MT: Matundu, MW: Mwanihana).

Parasite type	Forests*			
	MA (*n* = 66)	US (*n* = 44)	MT (*n* = 60)	MW (*n* = 81)
*Trichuris* sp.	39.4	63.6	43.3	60.5
strongylid nematode	7.6	29.5	3.3	37.0
*Strongyloides fulleborni*	15.2	13.6	28.3	18.5
*Strongyloides stercoralis*	0.0	4.5	0	0
*Colobenterobius* sp.	1.5	2.3	0	2.5

* Forests are ordered by protection level and degree of human impact, from least protected and most disturbed (MA forest) to most protected and least disturbed regarding human impact (MW forest).

Number of samples (*n*) analyzed per forest are expressed in brackets.

The prevalence of these parasites differed markedly with *Trichuris* sp. infecting more than 60% of individuals in two forests (MW and US) and *Colobenterobius* sp. infecting 2.5% of individuals (in MW; Table 2).

#### Parasite prevalence among forest blocks (model 1)

Parasite prevalence did not differ significantly among forests (likelihood ratio tests, *Trichuris* sp.: χ^2^ = 4.155, df = 3, P = 0.245; *S*. *fulleborni*: χ^2^ = 4.371, df = 3, P = 0.224; strongylid nematode: χ^2^ = 3.779, df = 3, P = 0.286). Furthermore, none of the three models revealed a significant seasonal variation in parasite prevalence (*Trichuris* sp.: χ^2^ = 2.422, df = 2, P = 0.298; *S*. *fulleborni*: χ^2^ = 5.616, df = 3, P = 0.060; strongylid nematode: χ^2^ = 0.847, df = 2, P = 0.655; Table 3).

**Table 3:** Results of models predicting parasite prevalence (response) across forests (MA: Magombera; US: Uzungwa Scarp; MT: Matundu, MW: Mwanihana).

Term^(1)^	Estimate	SE	lower Cl	upper Cl	min	max
*Trichuris* sp.						
Intercept	0.038	0.432	-0.817	0.976	-0.282	0.435
MT	-0.665	0.792	-2.455	0.844	-1.317	-0.213
MW	0.429	0.446	-0.462	1.334	0.233	0.984
US	0.474	0.585	-0.741	1.649	0.255	1.089
sin(date.rad)	0.074	0.456	-0.792	1.017	-0.119	0.537
cos(date.rad)	-0.493	0.323	-1.226	0.115	-0.788	-0.149
strongylid nematode						
intercept	-3.184	1.206	-7.699	-0.761	-4.083	-2.389
MT	-1.035	2.405	-19.536	3.773	-16.977	1.460
MW	1.660	1.458	-2.041	5.157	0.326	4.730
US	0.446	1.877	-4.446	5.275	-0.897	4.146
sin(date)	-1.157	1.439	-5.191	1.301	-1.779	1.751
cos(date)	-0.530	0.929	-2.901	1.212	-1.291	1.418
*Strogyloides fulleborni*						
Intercept	-2.667	0.693	-4.593	-1.457	-3.729	-2.318
MT	2.245	1.225	-0.677	5.251	1.652	4.129
MW	1.163	0.600	-0.143	2.430	0.882	1.767
US	1.367	0.872	-0.558	3.332	1.089	2.092
sin(date)	0.330	0.659	-1.227	1.713	-0.222	0.690
cos(date)	1.156	0.507	0.177	2.710	0.882	2.099

^(1)^ population was dummy coded with MA being the reference category; sin(date) and cos(date) model potential seasonal variation in parasite prevalence.

Estimates, together with standard error (SE), lower and upper confidence interval (Cl) and minimum (min) and maximum (max) of estimates obtained when excluding groups one at a time are reported.

#### Parasite richness among forest blocks (model 2)

**Fig 3:**
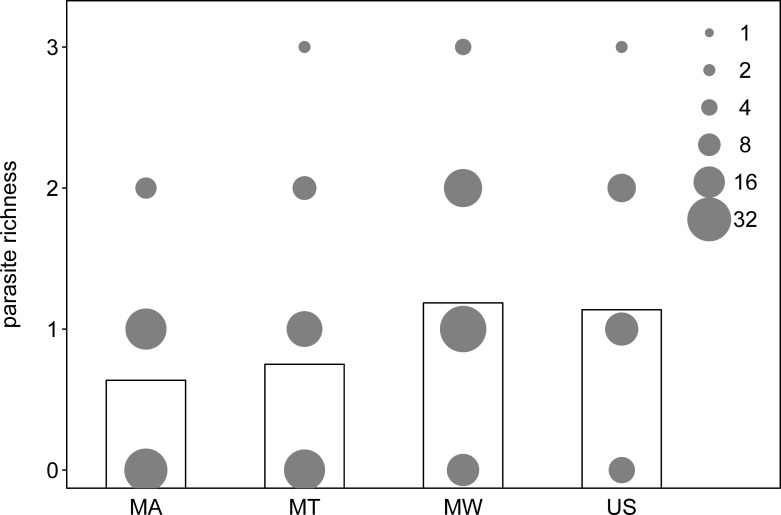
Parasite richness. Parasite richness for the four forests (MA: Magombera, US: Uzungwa Scarp Nature Reserve; MT: Matundu, MW: Mwanihana). The area of the dots corresponds to the number of samples with the respective parasite richness in each forest, and the bars indicate the average parasite richness per forest.

We found no significant differences in parasite richness among individuals of different forests (χ^2^ = 5.949, df = 3, P = 0.114). Moreover, the effect of season also appeared non-significant (χ^2^ = 0.158, df = 2, P = 0.924; Table 4, Fig 3).

**Table 4:** Results of the model predicting parasite richness (response) across forests (MA: Magombera; US: Uzungwa Scarp; MT: Matundu, MW: Mwanihana).

	Estimate	SE	lower Cl	upper Cl	min	max
Intercept^(1)^	-1.972	0.261	-2.556	-1.467	-2.330	-1.779
US	0.622	0.367	-0.177	1.380	0.482	1.535
MT	0.293	0.363	-0.396	1.103	-0.376	0.517
MW	0.727	0.285	0.130	1.295	0.495	1.485
sin(date)	-0.118	0.295	-0.754	0.430	-0.355	0.647
cos(date)	-0.011	0.182	-0.403	0.351	-0.151	0.452

^(1)^ population was dummy coded with MA being the reference category; sin(date) and cos(date) model potential seasonal variation in parasite richness

Estimate, together with standard error (SE), lower and upper confidence limit (Cl) and minimum (min) and maximum (max) of estimates obtained when excluding groups one at a time are reported.

#### Comparisons of groups within forests

Furthermore, across groups living in the same forest, we found clear differences in parasite prevalence only in part (estimated standard deviations for the contribution of the random effect of group, *Trichuris* sp.: <0.001.; *S*. *fulleborni*: 0.361; strongylid nematode: 1.486). In fact, most of these estimates were roughly of the same order of magnitude as the estimated fixed effects (Table 3), showing that variation among groups was about as large as variation due to differences among forests or seasonal variation. However, with regard to parasite richness, variation among groups was very low (0.0001).

#### Parasite richness as a function of altitude and stress hormones (model 3)

**Fig 4:**
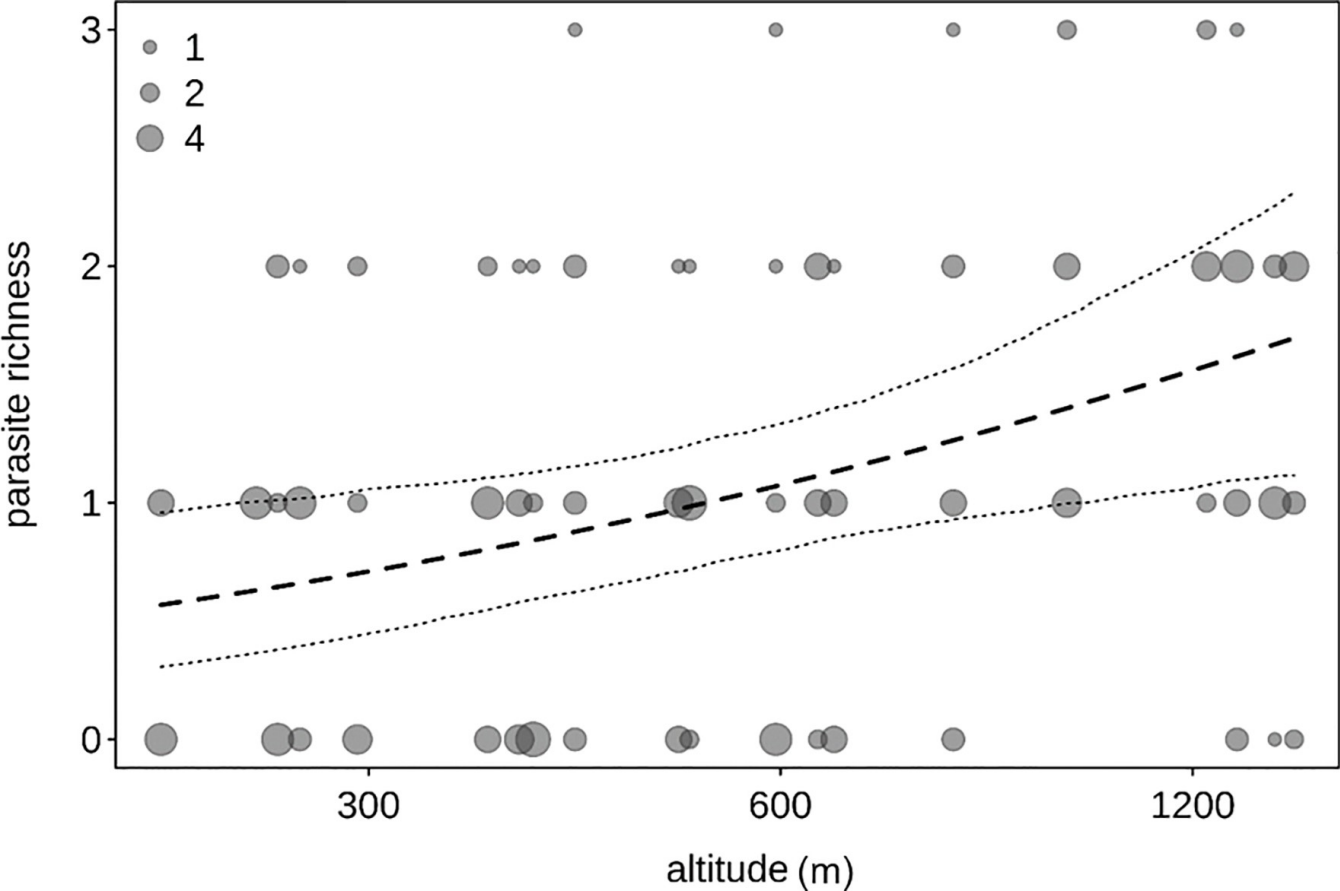
Parasite richness and altitude. Parasite richness as a function of altitude across the four forest blocks (MA: Magombera, US: Uzungwa Scarp Nature Reserve; MT: Matundu, MW: Mwanihana) within the Udzungwa Mountains in Tanzania. The area of the dots corresponds to the number of samples (from 1 to 7) per bin of altitude. The dashed line and dotted lines represent the fitted model and its confidence limits with all other predictors centered.

Overall, parasite richness was influenced by the factors investigated (full null comparison: χ^2^ = 7.727, df = 2, P = 0.021). More specifically, parasite richness increased with altitude (Table 5; Fig 4). Stress hormone levels was not significantly associated with parasite richness, nor were season or forest (Table 5).

**Table 5:** Results of the model predicting parasite richness (response) across forests (MA: Magombera; US: Uzungwa Scarp; MT: Matundu, MW: Mwanihana), including altitude and stress hormone (faecal glucocorticoid level, FGC) as additional predictors.

	Estimate	SE	lower Cl	upper Cl	χ^2^	df	P	min	max
Intercept	-2.064	0.537	-3.118	-1.051				-2.727	-1.726
FGC	0.010	0.022	-0.035	0.052	0.208	1	0.648	-0.004	0.031
altitude^(1)^	0.041	0.016	0.009	0.074	6.746	1	0.009	0.032	0.057
US	-0.316	0.444	-1.114	0.468				-0.719	0.469
MT^(2)^	-0.597	0.440	-1.508	0.222	5.256	3	0.154	-1.001	-0.201
MW	0.011	0.360	-0.648	0.687				-0.162	0.678
sin(date)^(3)^	0.233	0.313	-0.344	0.848	0.620	2	0.734	-0.116	0.772
cos(date)	-0.042	0.230	-0.510	0.412				-0.179	0.366

^(1)^ square root transformed after subtracting its minimum.

^(2)^ population was dummy coded with MA being the reference category; the indicated test refers to the overall effect population.

^(3)^ sin(date) and cos(date) model potential seasonal variation in parasite richness; the indicated test refers to the overall effect.

Estimate, standard error (SE), lower and upper confidence limit (Cl), results of significance tests, and minimum (min) and maximum (max) of estimates obtained when excluding groups one at a time are reported.

### Parasite prevalence as a function of altitude and stress hormones (model 4)

The results for the models estimating the effects of altitude and stress hormone levels on parasite prevalence depended on the taxon considered. While for two taxa the full null model comparison was not significant (*Trichuris* sp.: χ^2^ = 0.733, df = 2, P = 0.693; *S*. *fulleborni*: χ^2^ = 0.085, df = 2, P = 0.958), for the strongylid nematode it was clearly significant (χ^2^ = 14.448, df = 2, P = 0.001; Table 6).

**Table 6:** Results of the models predicting parasite prevalence (response) across forests (MA: Magombera; US: Uzungwa Scarp; MT: Matundu, MW: Mwanihana), including altitude and stress hormone (faecal glucocorticoid levels, FGC) as additional predictors.

	Estimate	SE	lower Cl	upper Cl	χ^2^	df	P	min	max
*Trichuris* sp.									
Intercept	2.030	1.544	-1.024	5.906				0.915	2.936
FGC^(1)^	-0.050	0.060	-0.186	0.073	0.703	1	0.402	-0.093	-0.020
altitude^(1)^	0.020	0.037	-0.054	0.099	0.303	1	0.582	0.000	0.053
MT^(2)^	-2.548	1.282	-6.224	-0.370	6.596		0.086	-3.439	-1.659
MW^(2)^	-0.102	0.764	-1.796	1.522		3		-0.625	1.418
US^(2)^	-0.725	0.958	-3.100	1.216				-1.212	0.900
sin(date)^(3)^	0.636	0.664	-0.627	2.310	5.460	2	0.065	0.256	1.648
cos(date)	-1.193	0.581	-2.862	-0.229				-1.538	-0.588
*Strongyloides fulleborni*									
Intercept	-2.160	1.719	-6.814	1.255				-4.861	0.449
FGC^(1)^	0.020	0.073	-0.132	0.167	0.073	1	0.787	-0.051	0.086
altitude^(1)^	-0.002	0.050	-0.113	0.120	0.002	1	0.968	-0.107	0.036
MT^(2)^	0.753	1.317	-2.285	4.863	0.655		0.884	-0.452	3.806
MW^(2)^	0.747	0.953	-1.504	2.769		3		-0.443	2.441
US^(2)^	0.806	1.110	-1.648	3.831				-0.483	2.476
sin(date)^(3)^	0.776	0.777	-0.937	2.835	1.484	2	0.476	-0.526	1.467
cos(date)	0.492	0.648	-0.697	2.886				-0.647	1.677
strongylid nematode									
Intercept	1.216	1.271	-8.079	7.862				-2.953	2.476
FGC^(^^1^^,^ ^4^^)^	-0.298	0.322	-1.220	0.370	0.775	1	0.379	-0.750	0.194
altitude^(^^1^^,^ ^5^^)^	3.323	0.826	2.137	10.361	13.438	1	*<0*.*001*	2.286	4.585
MT^(2)^	-3.337	1.894	-21.471	-0.089	7.866		0.049	-19.803	2.699
MW^(2)^	-3.025	1.597	-15.560	6.026		3		-4.905	0.254
US^(2)^	-4.698	1.875	-21.309	1.507				-8.053	-0.618
sin(date)^(3)^	0.706	1.320	-4.004	5.217	0.812	2	0.666	-1.942	3.189
cos(date)	0.837	0.883	-1.482	3.376				-0.198	3.480

^(1)^ square root transformed after subtracting its minimum.

^(2)^ population was dummy coded with MA being the reference category; the indicated test refers to the overall effect population.

^(3)^ sin(date) and cos(date) model potential seasonal variation in parasite prevalence; the indicated test refers to the overall effect.

^(4)^ z-transformed to a mean of zero and a standard deviation of one, mean and sd of the original variable were 21.102 and 3.487, respectively.

^(5)^ z-transformed to a mean of zero and a standard deviation of one, mean and sd of the original variable were 17.813 and 11.515, respectively.

Estimate, standard error (SE), lower and upper confidence limit (Cl), results of significance tests, and minimum (min) and maximum (max) of estimates obtained when excluding groups one at a time are reported.

**Fig 5:**
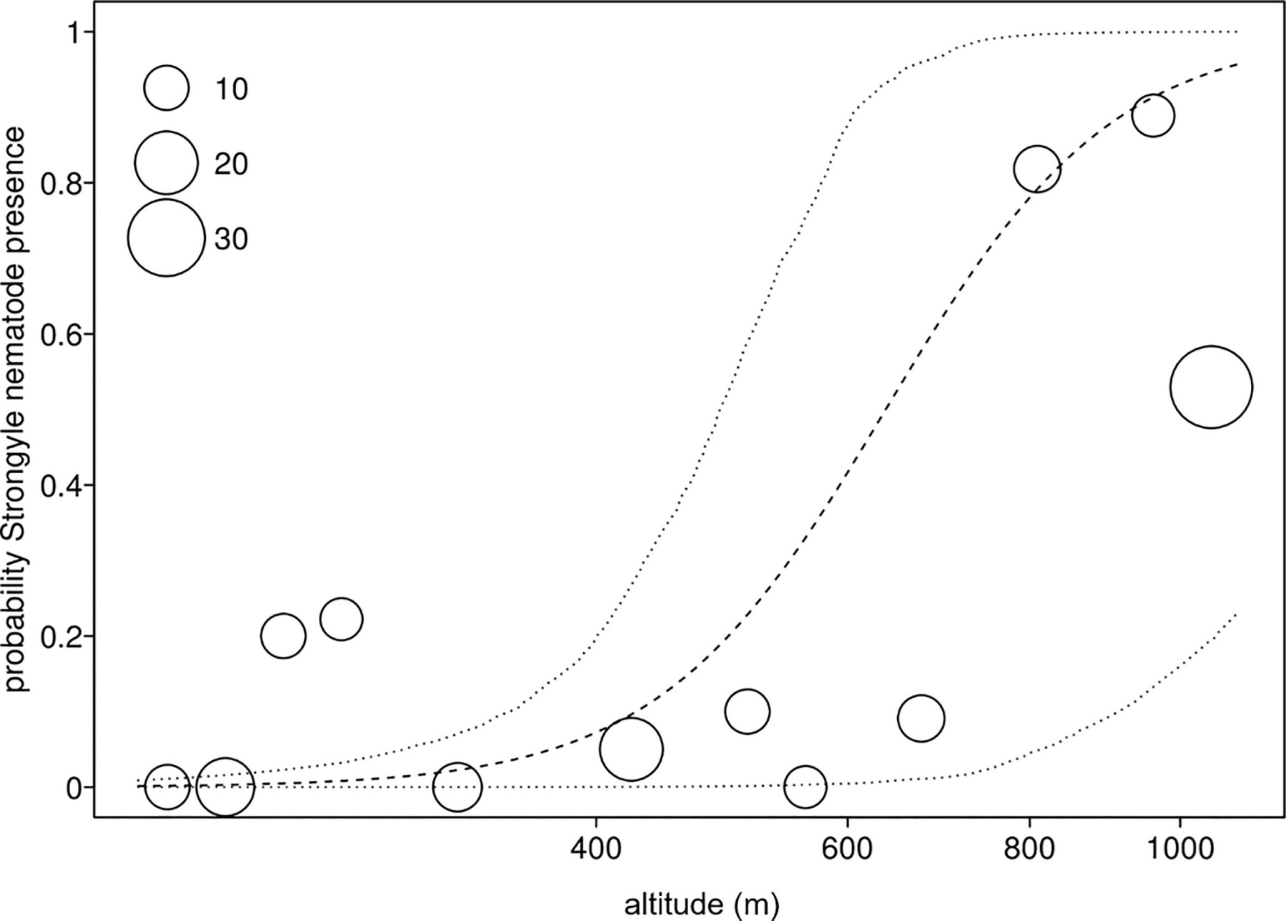
Strongylid nematode prevalence and altitude. Prevalence of the strongylid nematode as a function of altitude across the four forest blocks (MA: Magombera, US: Uzungwa Scarp Nature Reserve; MT: Matundu, MW: Mwanihana) within the Udzungwa Mountains in Tanzania. The area of the dots corresponds to the number of samples (from 9 to 34) per bin of altitude. Dashed and dotted lines represent the fitted model and its confidence limits with all other predictors centered.

In this model we found that the prevalence of the strongylid nematode increased with increasing altitude (Fig 5). Furthermore, the control predictor population revealed significance, while season did not have a significant effect in two of the three models but tended to have an impact on *Trichuris* sp. prevalence (Table 6).

## Discussion

Under the hypothesis that human activities contributing to habitat loss and degradation would also influence biodiversity at micro scales [9, 11, 16, 19, 65], we investigated gastrointestinal parasites of the endangered Udzungwa red colobus monkeys living in protected and human-impacted habitats. Despite the complexity of parasite infection risks and the potential limitation of the methods applied [66], from a comparison among forest types neither of the parasite indices examined (i.e., parasite prevalence and richness) significantly varied between fragmented, unprotected forests and intact, protected ones, as noted previously for other primate species [9, 11, 13, 15, 16, 67, 68]. However, considering that human activities are concentrated at forest edges (in both protected and unprotected forests) at lower altitudes [36, 69], the finding of a reduction in parasite richness at lower altitudes could still be explained as an indirect influence of human activities. Other studies have explained such reduction in parasite richness as a consequence of a higher diet quality in open, fragmented areas as in roe deer (*Capreolus capreolus*) populations [70], or in the coastal lowland Chacma baboons (*Papio cynocephalus ursinus*) compared to montane ones [71]. Indeed the reduction of nematodes in Australian skinks *Lampropholis guichenoti* was inferred to habitat disruption [72], as well as for some helminth species in the sigmodontinae rodent species (*Akodon cursor*, *A*. *montensis* and *Oligoryzomys*) from Brazil [73] and in a black howler monkey (*Alouatta pigra*) population [19]. Although the ecological importance of parasites and their critical roles in food webs and ecosystem processes have been largely discussed [74], still few data are available on the impact of habitat disturbance and parasite infections.

The five gastrointestinal parasites (all nematodes) identified have all been found in other red colobus species, such as Ugandan red colobus (*Piliocolobus tephrosceles*), eastern black-and-white colobus (*Colobus guereza*) and Angolan black-and-white colobus (*C*. *angolensis*) [9, 75]. However, in agreement with Altizer and colleagues [76] who propose that threatened species have lower richness of parasites than non-threatened species, Udzungwa red colobus host fewer parasite types than colobines of least concern (*Cercopithecus ascanius*: 10 parasite types [13], *Colobus vellerosus*: 11 [77]). Despite their predominant arboreality (with occasional descent to the ground), more than 70% of the Udzungwa red colobus individuals were infected with at least one nematode species. Thus, their arboreal behaviour habit does not prevent them from becoming infected with nematode eggs or larvae from the feces of other infected group members or other animals. Indeed, most individuals (52%) were infected with *Trichuris* sp. that does not need an intermediate host to develop, but are transmitted through the ingestion of eggs and passed via hosts’ feces onto soil, water and food items. Also *S*. *fulleborni* and the unidentified strongylid nematode were well represented in all four forests, as documented for other monkeys [16, 78] and apes [79]. However, because the identification of strongylid species based on the eggs’ morphology is difficult, discussing the potential impact of this unidentified species on host health is purely speculative. Although flotation and sedimentation methods are not the optimal methods to infer the prevalence of pinworms, *Colobenterobius* sp. were found in three out of four forests (MA, MW and US). Moreover, *S*. *stercoralis*, a common human parasite, rarely found in wild primates, infected individuals inhabiting one forest (US) where humans are often present because illegal hunting of primates is still practiced.

When considering the potential association between parasite infections and measures of stress levels [10, 11, 12], Udzungwa red colobus did not show any association between FGC levels and parasite infections. A lack of association could be due to the sampling method since sex and reproductive condition of individuals were unknown [80]. However, our findings showing no association between FGC and parasite infection may still suggest that the interaction between immune- and endocrine functions of the host are activated independently as also suggested in other primate (lemurs [19]; gibbons [30]; howler monkeys [32]; colobines [78]) and non-primate species (mouse [81]; raccoons [82]; African ungulates [83]). Moreover, it is possible that the lack of association is an artefact of our sampling design, since more frequent longitudinal sampling would be required to firmly establish both parasite infection and stress levels.

Indeed, models of parasitism in animals indicate that parasitic infections may be affected by climatic conditions [84] as well as geophysical characteristics of the environment as shown in non-human primates (e.g., howler monkeys [28], colobines [78], vervet monkeys [31] and lemurs [20]). Our finding of a positive association between altitude and parasite richness can be explained by the variation of the forest physical characteristics across an altitudinal gradient. In fact, it is likely that parameters such as humidity, water availability and canopy cover do change between habitats at different altitudes, making the contrasting habitats more suitable for parasites and thus facilitating host infections [82]. However, the unexpected result of a reduction in parasite richness in animals inhabiting forests at lower instead of higher altitude, made us formulate an alternative explanation. In the Udzungwas, human presence and activities are intensified at lower altitude in both protected and unprotected forests [35, 85]; such anthropogenic pressure may play an active role in shaping habitat features over time and consequently reducing parasite presence and potential transmission.

We cannot exclude that clearance of bigger trees during logging activities and consequent creation of forest edges change the forest structure, and thus parasite life cycles. For example, higher exposure to sun radiation and increased wind, due to reduced canopy cover in disturbed forests, may promote drier environments [86]. Based on these altered conditions, low humidity that reduces survival of free-living stages of parasites would be expected and consequently lower parasite infections in affected habitats. Moreover, the study areas at lower altitudes are surrounded by cultivated fields usually treated with pesticides, fertilizers and anti-helminthics resulting in a lower contamination of helminths, which is particularly true for the small lowland forest fragment (MA), surrounded by sugar cane plantations [87]. Thus, when all of this evidence is considered together, it appears that a combination of factors means that the results could still reflect habitat disturbance.

Finally, our findings of reduced helminth richness at lower altitude where human disturbance is higher agree with lower gut bacterial diversity found in the same animal populations inhabiting these forests [39]. In light of plausible interactions between helminths and bacteria in natural environments [88, 89], it cannot be excluded that such losses in micro-biodiversity could hamper the long-term viability of threatened populations. Thus, we recommend and foresee more detailed investigations to understand whether such interactions do exist.

## Supporting information

S1 Table Number of samples analyzed per group across forests (MA: Magombera; US: Uzungwa Scarp; MT: Matundu, MW: Mwanihana) on models 1 and 2 (N1) and models 3 and 4 (N2). (DOCX)
