# Correction: Altitude and human disturbance are associated with helminth diversity in an endangered primate, *Procolobus gordonorum*

**DOI:** 10.1371/journal.pone.0251617

**Published:** 2021-05-06

**Authors:** Claudia Barelli, Viviana Gonzalez-Astudillo, Roger Mundry, Francesco Rovero, Michael Heistermann, Heidi C. Hauffe, Thomas R. Gillespie

Michael Heistermann is not included in the author byline. Michael Heistermann should be listed as the fifth author and affiliated with Endocrinology Laboratory, German Primate Center, Leibniz Institute for Primate Research, Göttingen, Germany. The contributions of this author are as follows: data curation. The correct is: Barelli C, Gonzalez-Astudillo V, Mundry R, Rovero F, Heistermann M, Hauffe HC, et al (2019) Altitude and human disturbance are associated with helminth diversity in an endangered primate, Procolobus gordonorum. PLoS ONE 14(12): e0225142. https://doi.org/10.1371/journal.pone.0225142

In the Assessment of stress hormone levels subsection of the Materials and methods section, the authors would like to add the following paragraph: FGC concentrations in the fecal extracts were determined using a group-specific enzyme immunoassay for the measurement of 5-reduced 3alpha, 11ß-dihydroxylated cortisol metabolites according to the method described earlier [38]. All samples were run in duplicate, and samples with a coefficient of variation of >7% between duplicates were re-measured. Sensitivity of the assay was 1 pg. Intra- and inter-assay coefficients of variation of high- and low-value quality controls were 6.2% and 7.6% and 11.3% and 13.1%, respectively.

## References

[1] Barelli C, Gonzalez-Astudillo V, Mundry R, Rovero F, Hauffe HC, Gillespie TR (2019) Altitude and human disturbance are associated with helminth diversity in an endangered primate, *Procolobus gordonorum*. PLoS ONE 14(12): e0225142. 10.1371/journal.pone.0225142 31800582 PMC6892551

