# Supplementary material for: Altitude and human disturbance are associated with helminth diversity in an endangered primate, Procolobus gordonorum
Source: PLoS One. 2019 Dec 4;14(12):e0225142. doi: 10.1371/journal.pone.0225142 (PMC6892551; doi:10.1371/journal.pone.0225142)
Supplement: S1 Table — (DOCX) [file pone.0225142.s001.docx]

**S1 Table**. **Number of samples analyzed per group across forests (MA: Magombera; US: Uzungwa Scarp; MT: Matundu, MW: Mwanihana) on models 1 and 2 (N1) and models 3 and 4 (N2).**

|  | **Date** | **Group** | **N 1** | **N 2** |
| --- | --- | --- | --- | --- |
| 1 | 28/11/2011 | MA 11 | 10 | 10 |
| 2 | 29/11/2011 | MA 13 | 10 |  |
| 3 | 30/11/2011 | MA 14 | 11 | 11 |
| 4 | 01/12/2011 | MA 15 | 10 |  |
| 5 | 09/12/2011 | MA 17 | 10 | 10 |
| 6 | 20/12/2011 | MA 22 | 6 | 6 |
| 7 | 22/12/2011 | MA 25 | 9 | 9 |
| 1 | 21/08/2012 | US 1 | 10 | 10 |
| 2 | 13/09/2012 | US 2 | 11 | 11 |
| 3 | 01/10/2012 | US 4 | 9 | 9 |
| 4 | 29/10/2012 | US 6 | 14 | 14 |
| 1 | 06/02/2012 | MT 11 | 10 | 10 |
| 2 | 08/02/2012 | MT 15 | 10 | 10 |
| 3 | 23/02/2012 | MT 17 | 10 | 10 |
| 4 | 16/01/2012 | MT 2 | 10 |  |
| 5 | 17/01/2012 | MT 3 | 10 | 10 |
| 6 | 21/01/2012 | MT 8 | 10 | 10 |
| 1 | 06/07/2011 | MW 4 | 9 |  |
| 2 | 20/07/2011 | MW 11 | 9 | 9 |
| 3 | 23/08/2011 | MW 20 | 10 |  |
| 4 | 20/09/2011 | MW 25 | 10 | 10 |
| 5 | 08/10/2011 | MW 32 | 10 | 10 |
| 6 | 24/10/2011 | MW 34 | 10 | 10 |
| 7 | 11/11/2011 | MW 39 | 11 | 11 |
| 8 | 05/01/2012 | MW 40 | 12 | 12 |
|  |  | 25 (20) | 251 | 202 |
